# Exploring the agronomic traits, antioxidant and antifungal properties of *Hermetia illucens* frass extract in durum wheat (*Triticum durum* Desf.)

**DOI:** 10.1186/s12870-025-07086-5

**Published:** 2025-08-14

**Authors:** Antonella Vitti, Leonardo Coviello, Patrizia Falabella, Stefania Mirela Mang, Carmen Scieuzo, Francesco Iannielli, Domenico Ronga, Maria Nuzzaci

**Affiliations:** 1 Department of Agricultural, Forestry, Food and Environmental Sciences, University of Basilicata, Via dell’Ateneo Lucano 10, 85100 Potenza, Italy; 2 Pharmacy Department, University of Salerno, Via Giovanni Paolo II, 132, 84084 Fisciano, Italy; 3 Department of Basic and Applied Sciences, University of Basilicata, Via dell’Ateneo Lucano 10, 85100 Potenza, Italy; 4 Spinoff XFlies s.r.l, University of Basilicata, Via dell’Ateneo Lucano 10, 85100 Potenza, Italy

**Keywords:** *Triticum durum* Desf. var *Simeto*, *Trichoderma afroharzianum* strain T22, *Fusarium sporotrichioides*, Seed priming, *Paenibacillus polymyxa*, *Botrytis cinerea*, *Fusarium oxysporum* f.sp. *lycopersici*

## Abstract

**Background:**

Wheat (*Triticum* spp.), the most cultivated species worldwide, is threatened by various stresses. Among these, the biotic stresses caused by phytopathogenic fungi, like *Fusarium sporotrichioides*, are responsible for food losses and mycotoxins poisoning. The green strategy based on recovery and use of frass deriving from *Hermetia illucens* reared on the standard Gainesville diet was applied, involving durum wheat (*Triticum durum* Desf. var *Simeto*) seed priming with 10% frass extract, alone or combined with *T. afroharzianum* T22 (T22), in pot/soil experiment. For this purpose, the agronomic traits, reduction of damping-off due to *F. sporotrichioides*, and activity of the pool of antioxidant enzymes involved were evaluated. In addition, the presence of microorganisms in the frass extract with possible plant growth promoting and/or protection activity, was searched.

**Results:**

Seed priming determined enhanced wheat growth performance and, in the meantime, a control of the development of disease symptoms, allowing a reduction of damping-off of almost 40% when frass extract and T22 were used together. This was accompanied by an increased antioxidant activity in seedlings derived from primed seeds, enabling them to face stresses in a proper way. In addition, in order to address which component of frass extract was responsible for these effects, *Paenibacillus polymyxa* was isolated from frass extract, and tested for its antifungal activity in vitro, resulting effective against *F. sporotrichioides* and also the phytopathogenic fungi *Fusarium oxysporum* f.sp. *lycopersici* and *Botrytis cinerea*.

**Conclusions:**

This finding demonstrated that seed priming with frass extract, together with *T. afroharzianum* T22, could be used as an effective and environmentally friendly strategy to promote wheat growth and, at the same time, effectively control the development of *F. sporotrichioides* disease. The insights gathered from this research, confirmed the ability of frass to be used in priming technique, opening the door to promising solutions to harness the potential of sustainable agricultural practices and green technologies circular economy-based.

## Background

The insect *Hermetia illucens*, belonging to Diptera order and Stratiomyidae family, known as the black soldier fly (BSF), is commonly used worldwide as model to produce high value products through the bioconversion process, which is highly sustainable [1]. Indeed, BSF larvae have the ability to convert every type of low value organic by-products, like crop residue, agri-food waste, or even animal manure [2–4] in insect biomass, very rich in fat and proteins mainly used as animal feed [5]. At the end of the bioconversion process, it is possible to obtain a very promising and valuable waste product from the BSF rearing, called frass. These are a mixture of larval excrement, undigested organic waste and shed exoskeletons that are produced during the bioconversion process [6, 7]. Frass could be useful in various industrial processes like biogas production [8], but due to the balanced organic composition represents also a viable alternative to commercial fertilizers with beneficial effects on different crops [9].

Wheat (*Triticum* spp.) represents the main nourishment for a large part of the world’s population [10]. Overall, it is the most widely cultivated species worldwide, even surpassing rice in terms of harvested area in 2022 [11]. Wheat is a strategic crop for food safety as it is a key source of dietary calories (worldwide, approximately 20% of calories and 55% of carbohydrates are provided by wheat). Durum wheat (*Triticum durum* Desf.) is widely cultivated in the Mediterranean area, and studies carried out with both ancient and modern varieties have provided valuable information on the characteristics of the development of the root system when a biostimulant is used [12], even under use of biotic stress conditions [13]. Climate change, associated with rising global temperatures and extremes in rainfalls, is significantly impacting wheat production in many parts of the world [14]. Biotic stresses, like *Fusarium* head blight (FHB) disease, caused by more than twenty fungal species belonging to *Fusarium* genus, are responsible for significant yield losses, and are also able to produce mycotoxins [15], in particular *F. sporotrichioides* [16, 17]. Mycotoxins content in grain induced by this pathogen, and *F. sporotrichioides* itself, can be reduced by the application of fungicides, but also using some biocontrol agents (BCAs). Among BCAs, *Trichoderma* spp., opportunistic plant symbionts, are fungi commonly isolated from soil and rhizosphere, and possess the ability to control soil-borne pathogens, playing a key role in resistance induction in planta [18], and also to enhance plant growth [13, 15, 19–21]. The systemic response induced by *Trichoderma* spp. has been observed on a wide range of species and may be temporally and spatially distant from the time and site of inoculation [18, 22, 23]. Wiśniewska et al. (2011) [15] found that *T. harzianum* AN4 has been useful in the control of *F. sporotrichioides*, by reducing its growth and inhibiting mycotoxins accumulation in grain.

In a former study [24], pasteurized frass extract deriving from BSF larvae demonstrated the potential to be used as a sustainable tool to induce biostimulation and antifungal activity in *Triticum durum* Desf. var *Simeto* against the soil-borne pathogen *F. sporotrichioides*, also in combination with the known BCA *Trichoderma afroharzianum* T22, by the priming treatment of seeds. The disease reduction has been attributed to both enzymatic and non-enzymatic responses, because differences in total phenolic content and superoxide dismutase activity (SOD) in seedlings derived from treated seeds have been observed. Effectively, a clear evidence of the biotic stresses perception by the plant is the overproduction of reactive oxygen species (ROS), which cause oxidative stress and whose detoxification by antioxidant enzymes like catalase (CAT) and SOD, is essential to ensure plant production [25]. As a consequence, the regulation of the activity of the pool of antioxidant enzymes is crucial to fine-tune the adaptation mechanisms in conditions of oxidative stress induced by phytopathogens. In another study, the ability of frass deriving from the insect *Tenebrio molitor* to promote the tolerance of chard plants against various stresses has been demonstrated and attributed to the presence of microorganisms in the frass with growth promoting activity [26]. Furthermore, the application of frass to potted soil has been able to influence the plant-associated soil microbial communities stronger than a conventional compost used as fertilizer [27].

In continuation to the above-mentioned work [24], the aim of the present study was to further evaluate the ability of frass as a green and sustainable strategy, by moving from a plate system studied during our first research, to a system that involved germination in soil. For this purpose, the effect of frass extract, alone or combined with *T. afroharzianum* T22, on potted wheat seedlings derived from primed seeds, was evaluated in terms of agronomic traits, reduction of damping-off due to *F. sporotrichioides*, and activity of the pool of antioxidant enzymes involved. In addition, the presence of microorganisms in the frass extract with possible plant growth promoting and/or protection activity, was searched in order to obtain, for the first time, a possible biological relevance of the frass extract to be employed for seed priming in sustainable agricultural systems.

## Methods

### Plant material, frass extract from *Hermetia illucens*, and fungal isolates

Seeds of durum wheat (*Triticum durum* Desf. var *Simeto*) were considered. This variety consists in an Italian tetraploid modern durum wheat released in 1988 (Capeiti-8/Valnova), and was obtained by mutagenesis and crosses involving old wheat materials. Seeds were surface sterilized for 2 min in 1% sodium hypochlorite solution, and rinsed three times with sterile distilled water (dH_2_O).

Frass were provided by Xflies s.r.l. (Potenza, Italy) starting from *Hermetia illucens* hatched eggs, and neonates reared on the standard Gainesville diet (Mangimi Losasso s.r.l.—Balvano, Potenza, Italy), according to Coviello et al. (2024) [24].

Frass aqueous extract was prepared according to Coviello et al. 2024 [24]. Briefly, solid frass was pasteurized at 70 °C for 1 h, according to EC Regulation No 2021/1925 [28], and frass extract (pFE) was prepared adding 10 g of pasteurized frass in 100 mL (1:10 w/V) of sterile 0.5% NaCl physiological saline solution. After incubation on an orbital shaker at 150 rpm and 27 °C, the suspension was ultracentrifugated at 6,000 × *g* (15 min). Finally, the supernatant was recovered by filtering through a double layer of sterile gauze.

*Fusarium sporotrichioides* PZ2 strain (FS) was provided by Prof. Antonio Ippolito of Department of Soil, Plant and Food Sciences, University “Aldo Moro”, Bari, Italy.

*Trianum P* (Koppert Italia S.r.l., Viale delle Nazioni 7, Bussolengo, Verona, Italy) was used as source of *Trichoderma harzianum* strain T22 (T22), renamed *T. afroharzianum*, according to Chaverri et al. (2015) [29].

*Botrytis cinerea* isolate (BC) was supplied from the fungal collection of DAFE (Department of Agricultural, Forestry, Food and Environmental Sciences, University of Basilicata, Potenza, Italy).

*Fusarium oxysporum* f.sp. *lycopersici* (FOLYC) was provided by Dr. Catello Pane, Consiglio per la Ricerca in Agricoltura e L’analisi dell’Economia Agraria (CREA), Centro di Ricerca Orticoltura e Florovivaismo, Pontecagnano Faiano, Salerno, Italy.

Fungal mycelia were grown on potato dextrose agar (PDA) medium for 7 days in the dark at 23–26 °C.

### Experimental design

#### Seed priming with frass extract and *T. afroharzianum* and infection with *F. sporotrichioides*

Sterile seeds were primed with different solutions following the procedure described in Coviello et al. 2024 [24]. Priming solutions were constituted from pasteurized frass extract (pFE) diluted at 10% in dH_2_O (v/v) and/or spore suspension of *T. afroharzianum* T22 (T22) at 1 × 10^6^ conidia mL^-1^. The infection suspension was prepared using spores of *F. sporotrichioides* at 1 × 10^6^ conidia mL^-1^. The resulting four or five theses for biostimulation (1–4) and protective (1, 5–8) trials, respectively, were: (1) untreated and not infected (control CTRL); (2) frass extract (pFE); (3) *T. afroharzianum* (T22); (4) frass extract and *T. afroharzianum* (pFE + T22); (5) *F. sporotrichioides* (FS); (6) frass extract and *F. sporotrichioides* (pFE vs. FS); (7) *T. afroharzianum* and *F. sporotrichioides* (T22 vs. FS); (8) frass extract, *T. afroharzianum* and *F. sporotrichioides* (pFE + T22 vs. FS).

The presence of FS and/or T22 on the coated seed of each thesis was verified by fungus re-isolation on PDA medium, and mycelium and conidia characterization under optical microscope observation.

#### Growth conditions and agronomic and physiological analyses

Twenty primed seeds were sown in aluminum trays (210 × 280 × 60 mm, L×W×D) filled with autoclaved (at 121 °C for 30 min on two consecutive days) soil substrates (COMPO SANA^®^ Universal Potting Soil, COMPO Italia s.r.l., Italy) and arranged in complete randomized design with four replicates for each experiment. Throughout the experiment, seedlings were kept in a growth chamber with a 16/8 h photoperiod, light/dark (average T of 22 °C; average relative humidity of 60%), and tap watered every 3 days until the field water capacity was reached. The emergence was monitored daily. At 21 days after sowing (DAS), seed germination was evaluated by counting the number of germinated seeds for each tray, and the height, measured from ground level to the tip of the apical shoot for each seedling, was recorded. The SPAD index (SPAD-502 Chlorophyll meter, Konica Minolta Sensing Europe B.V., Cinisello Balsamo, MI, Italy) was measured on the fully expanded leaves with three measurements per plant. Seedlings were carefully removed from the tray, soaked in water to remove root attached substrate particles, dried with paper towels to remove excess water and separated into shoot and root portions. The length of the longest root of each seedling was measured; the fresh and dry weights (oven-dried at 60 °C until constant weight) of the shoots and roots were recorded.

Seed Germination Index (SGI) was calculated according to Vitti et al. (2024) [30]:

$$ \begin{gathered} {\text{SGI}}\% \hfill \\ = [({\text{n}}{\text{. seeds}}\,{\text{germinated}}\,{\text{in}}\,{\text{sample}}/{\text{n}}{\text{.seeds}}\,{\text{germinated}}\,{\text{in}}\,{\text{control}})] \hfill \\ \times 100 \hfill \\ \end{gathered} $$

#### Effect of seed treatment on damping-off caused by *F. sporotrichioides*

The presence of FS and/or T22 on the wheat root was verified at the end of the survey (21 DAS) by re-isolating the fungi on PDA and observing their mycelia and conidia under optical microscope. The effect of pFE and T22, both alone and combined, was evaluated as damping-off (DO) caused by FS on the emerged seedlings, according to the formula of Veeken et al. (2005) [31]:

$$ {\text{\% DO = [(HPo}}\, - \,{\text{HPi)}}\,{\text{/}}\,{\text{HPo]}}\, \times \,{\text{100}}\, $$

where: HPo is the number of healthy seedlings under the untreated experimental condition and HPi is the number of healthy seedlings derived by treated seeds and inoculated with FS.

#### Total antioxidant activity

Total antioxidant capacity was assessed using the Antioxidant Assay Kit (item No. 709001, Cayman Chemical, Ann Arbour, MI, USA) according to the manufacturer’s instructions. The kit allows to assess the antioxidant activity of both aqueous- and lipid-soluble antioxidants and relies on the ability of those molecules to inhibit the oxidation of ABTS to ABTS˙⁺ by metmyoglobin. Four tissue samples replicates were made for each thesis (*n* = 4) deriving by 500 mg of frozen tissues (leaf + root) from five seedlings, randomly chosen from each tray. The samples were homogenized in 5 mL of cold 5 mM potassium phosphate, pH 7.4 containing 0.9% sodium chloride and 0.1% glucose, and centrifuged at 10000 × *g* for 15 min at 4 °C. The resulting supernatant was recovered and absorbance was read at 750 nm with a microplate reader model Multiskan FC (Thermo Scientific, Fisher Scientific, Segrate, Italy). Antioxidant molecules in the sample caused a suppression of absorbance proportional to their concentration and were expressed as mM Trolox equivalents g^-1^ FW.

### Isolation, identification and antifungal activity of microorganisms derived from frass extract

#### Microorganisms isolation from pFE

Fifty µL of pFE were spread on Petri plate containing Luria-Bertani Agar (LBA) and PDA media for bacterial or fungal isolation, respectively. The plates were incubated in the dark at 30 °C for 24 h (for bacteria) or between 23 and 26 °C for 72 h (for fungi). No fungal colonies were observed after incubation.

The species resembling bacteria was isolated and grown on nutrient broth agar with glycerin (NGA) medium. Subcultures were performed by transferring the bacteria on the same medium to obtain pure cultures.

#### Molecular identification of the bacterial isolate

The pure bacterial cultures were cultivated overnight in LB liquid medium at 30 °C and under agitation (150 rev/min). Subsequently, they were aliquoted and conserved at -80 °C in 15% w/v glycerol (Merck KgaA, Darmstadt, Germany).

The total genomic DNA (gDNA) was extracted from nine single pure cultures using the DNeasy Plant Mini Kit (Qiagen, Hilden, Germany) following manufacturer’s instructions with minor modifications as described by Camele and Mang (2019) [32] and Mentana et al. (2019) [33]. The quantity and quality of the genetic material were checked by readings at ND-1000 spectrophotometer (Thermo Fisher Sci. Inc., MA, USA) and the gDNA was kept at -20 °C until further use. The extracted gDNA was amplified with three pairs of primers: the fD1 and rD1 amplifying a fragment of the *16 S rRNA* gene [34]; the gyrA-42 F and gyrA-1066r amplifying a fragment of the *gyrA* gene [35] and the rpoB-2292f and rpoB-3354r amplifying a portion of the *rpoB* gene [35] using the Phire Plant Direct PCR Master Mix (Thermo Scientific Inc., USA) and the protocols fully described in previous studies by Frisullo et al. (2015) [36] and Mang et al. (2022) [37]. PCR outcomes were observed by electrophoresis in a 1.2% agarose gel run at 50 V in Tris-acetate-EDTA (TAE) 1X running buffer. The amplicons were directly sequenced, in both directions, using the same primers as for the PCR assay. The obtained nucleotide sequences were aligned against the National Center for Biotechnology Information (NCBI) database using the Basic Local Alignment Search Tool (BLAST) program and the option Blastn for precise species identification.

#### Phylogenetic analysis of the bacterial isolate

The *16 S rRNA*, *gyrA* and *rpoB* partial genes sequences were first aligned by the ClustalW program in the MEGA11 phylogeny package, and the alignments were manually corrected [38]. Only the single barcode phylogenetic analysis using Maximum Likelihood (ML) method and Kimura-2 model [39] was performed, by the same phylogeny package, due to different isolates/strains (for each DNA barcode) data availability in the NCBI database. Stability of trees’ branches was tested by bootstrap analysis with 1000 replicates [40].

#### In vitro antagonistic effect of *Paenibacillus polymyxa* against phytopathogenic fungi

The inhibitory effect of bacterial isolate was evaluated according to Jiang et al. (2014) [41] with slight modifications. Conidial suspension of phytopathogenic fungi (*Botrytis cinerea*, *F. sporotrichioides* and *F. oxysporum* f.sp. *lycopersici*) was obtained by flooding a 7 days old PDA culture plate with dH_2_O. The suspension was filtered through double layered sterile gauze to remove mycelium and the conidial concentration was adjusted to 10^6^ conidia mL^-1^ using a hemocytometer (Thoma counting chamber, BLAUBRAND^(R)^, Wertheim, Germany).

Forty µl of conidial suspension of each phytopathogenic fungus were spread on a 90 mm PDA Petri plate. Thereafter, an 8 mm diameter well was made in the center of the plate, and 100 µl of *P. polymyxa* liquid culture or sterile LB medium (as control) were added to the well. The fungal growth inhibition was calculated after 72 h of incubation at 26 °C by subtracting the average of two diameters (90° from each other) of the zone around the well where no fungal growth was observed from the diameter of the fungal growth seen in the control Petri dish.

The experiment was carried out in triplicate.

### Statistical analysis

Normal distribution of data was tested by the Shapiro-Wilk test at *p* < 0.05 and homoscedasticity was tested performing the Breusch-Pagan test (*p* < 0.05). When the assumptions were not met, the variable was *z* transformed or subjected to log_10_ (count data). Data from agronomic and physiological analyses, indicated as percentages relative to the control (%), damping-off, antioxidant activity, and in vitro data, were expressed as mean (*n* = 4 or *n* = 3 for the in vitro test) ± SD and analyzed according to one-way ANOVA followed by Tukey’s HSD test (*p* < 0.05). Data from the in vivo experiment were subjected to principal component analysis (PCA) using the “PCA” function from “FactoMineR” package, considering an *eigenvalue* > 1 as cutoff point for which PC was retained [42]. R (version 4.2.3, R Foundation for Statistical Computing, Vienna, Austria) with the software RStudio IDE (release 2023.06.0 + 421) and packages Tidyverse [43] and MultcompView [44], to write and run R code were used.

## Results

### Effect of seed treatment on agronomic traits

In order to gain specific information regarding the effect on growth parameters by the treatments as such or during the interaction also with the pathogen, we have considered separately the analysis of the agronomic traits, in presence or not of the infection.

**Fig. 1:**
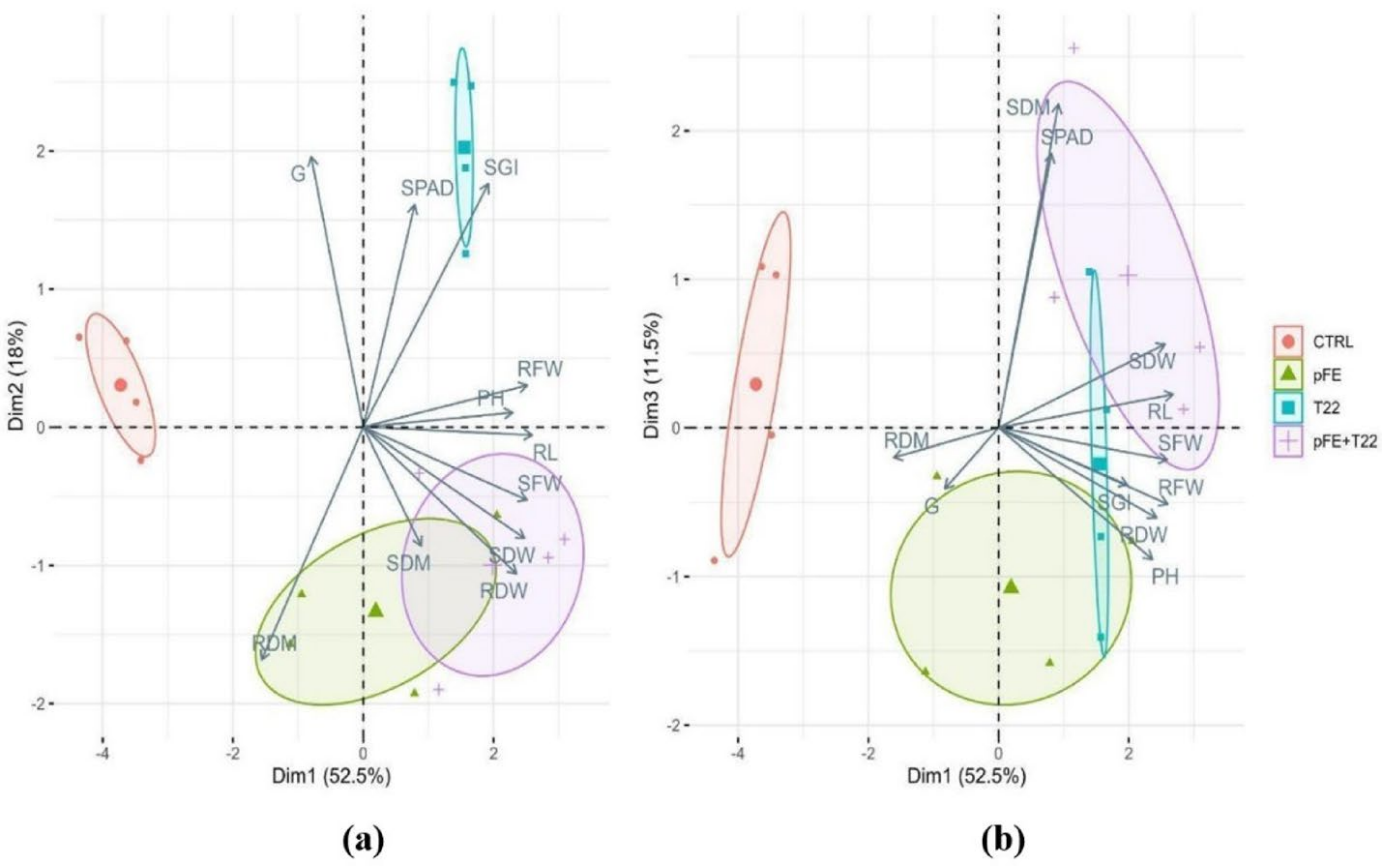
Biplots for the principal component analysis (PCA) outputs for the effect on growth parameters in durum wheat (*Triticum durum* Desf. var *Simeto*) seedlings derived from: untreated seed (CTRL) or seeds primed with frass extract (pFE) and *T. afroharzianum* T22 (T22), alone or together (pFE + T22). **a**: biplots of dimension 1 (Dim1) and dimension 2 (Dim2); **b**: biplots of dimension 1 (Dim1) and dimension 3 (Dim3). G = percentage of germinated seed/total sowed seeds (%), RL = radicle length (cm), SGI = seed germination index (%), PH = plant height (cm), SPAD = index of chlorophyll content in leaf, SFW = shoot fresh weight (g), RFW = root fresh weight (g), SDW = shoot dry weight (g), RDW = root dry weight (g), SDM = shoot dry matter (%), RDM = root dry matter (%)

The biostimulation effect induced by priming with pFE, T22 and pFE + T22 on wheat seeds treated, but not infected, is summarized in Fig. 1; Table 1.

Dimension 1 in PCA plot (Dim1) accounted for 52.5% of the variance, dimension 2 (Dim2) accounted for 18%, dimension 3 (Dim3) accounted for 11.5%, and their sum explained 82% of total variance. Dim1 was principally driven by root length (RL), plant height (PH), shoot fresh weight (SFW), root fresh weight (RFW), shoot dry weight (SDW), root dry weight (RDW). Dim1 clearly separated the control (CTRL) from all the other treatments, because CTRL was placed in the negative quadrant and characterized by significantly lower values of all above mentioned parameters (Table 1). On the contrary, pFE was the only one positioned across the two quadrants because it was not positively associated with high values of RL (Table 1). Differently from Dim1, Dim2 showed strong connection with the percentage of germinated seeds (G), SPAD, seed germination index (SGI) and root dry matter (RDM), with a negative relation between SPAD and SGI with RDM (Fig. 1a). Dim2 clearly separated both treatments with pFE and pFE + T22 from control but also from T22. In fact, this latter was entirely placed in the positive quadrant, while CTRL was across the axis, and pFE and pFE + T22 were in the negative quadrant. As shown in Fig. 1b, Dim3 was principally driven by shoot dry matter (SDM) and SPAD, that were strongly related to each other since their vectors were overlapped (Fig. 1b). Because of a positive association with its high values of SPAD and SDM, this latter being significantly different from the control, the pFE + T22 was the only treatment placed almost completely in the positive quadrant (Fig. 1b; Table 1). On the contrary, pFE was positioned on the negative quadrant of Dim3 due to the negative association with high values of both SPAD and SDM, although never significantly different from those of control (Fig. 1b; Table 1).

**Table 1:** Effect on growth parameters in durum wheat (*Triticum durum* desf. Var *Simeto*) seedlings derived from: untreated seed (CTRL) or seeds primed with Frass extract (pFE) and *T. afroharzianum* T22 (T22), alone or together (pFE + T22)

Treatment	G (%)	SW *p*	RL (cm)	SW *p*	SGI (%)	SW *p*	PH (cm)	SW *p*	SPAD	SW *p*
CTRL	95.00 ± 10.00 a	0.082	9.27 ± 1.14 c	0.564	95.38 ± 7.29 c	0.929	25.23 ± 2.22 b	0.955	37.17 ± 1.96 a	0.373
pFE	75.00 ± 10.20 b	0.051	13.73 ± 1.43 b	0.557	112.56 ± 12.98 bc	0.299	29.72 ± 3.51 ab	0.507	33.25 ± 4.40 a	0.132
T22	100.00 ± 0.00 a	0.442	16.34 ± 1.81 ab	0.300	181.77 ± 16.38 a	0.602	30.41 ± 0.97 a	0.389	39.63 ± 4.12 a	0.256
pFE + T22	70.20 ± 11.58 b	0.355	18.08 ± 2.21 a	0.751	129.52 ± 15.91 b	0.828	30.31 ± 1.99 a	0.494	39.58 ± 1.53 a	0.073
Statistics	df = 3, 12; F = 10.40; *p* < 0.01*; BP *p* = 0.183		df = 3, 12; F = 20.43; *p* < 0.01*; BP *p* = 0.183		df = 3, 12; F = 30.02; *p* < 0.01*; BP *p* = 0.183		df = 3, 12; F = 4.44; *p* = 0.03*; BP *p* = 0.183		df = 3, 12; F = 3.38; *p* = 0.05; BP *p* = 0.183	

Treatment	SFW (g)	SW p	RFW (g)	SW p	SDW (g)	SW p	RDW (g)	SW p	SDM (%)	SW p	RDM (%)	SW p
CTRL	0.48 ± 0.05 b	0.785	0.11 ± 0.05 b	0.677	0.057 ± 0.007 b	0.246	0.017 ± 0.003 b	0.789	11.601 ± 0.445 b	0.287	18.00 ± 2.0 a	0.383
pFE	0.65 ± 0.08 a	0.304	0.34 ± 0.05 a	0.499	0.077 ± 0.01 a	0.436	0.051 ± 0.008 a	0.568	11.642 ± 0.382 b	0.397	17.00 ± 2.0 ab	0.107
T22	0.66 ± 0.06 a	0.571	0.41 ± 0.03 a	0.311	0.077 ± 0.005 a	0.885	0.041 ± 0.003 a	0.282	11.747 ± 1.112 ab	0.060	12.00 ± 2.0 b	0.943
pFE + T22	0.75 ± 0.12 a	0.118	0.34 ± 0.03 a	0.698	0.092 ± 0.011 a	0.385	0.048 ± 0.004 a	0.994	12.651 ± 1.132 a	0.086	16.00 ± 4.0 ab	0.578
Statistics	df = 3, 12; F = 7.10; *p* < 0.01*; BP *p* = 0.183		df = 3, 12; F = 39.68; *p* < 0.01*; BP *p* = 0.183		df = 3, 12; F = 11.15; *p* < 0.01*; BP *p* = 0.183		df = 3, 12; F = 35.61; *p* < 0.01*; BP *p* = 0.183		df = 3, 12; F = 4.49; *p* = 0.02*; BP *p* = 0.183		df = 3, 12; F = 4.86; *p* = 0.02*; BP *p* = 0.183	

*G* Percentage of germinated seeds/total sowed seeds, *RL* Radicle length, *SGI * Seed germination index, *PH* Plant height, *SPAD* Index of chlorophyll content in leaf, *SFW*  Shoot fresh weight, *RFW* Root fresh weight, *SDW*  Shoot dry weight, *RDW* Root dry weight, *SDM* Shoot dry matter, *RDM* Root dry matter.

Different letters indicate significant differences between values, according to one-way ANOVA followed by Tukey post-hoc test at *p* < 0.05.

Data are expressed as the mean of 4 replicates (each of 20 seeds) ± SD. df = degree of freedom (treatment, residuals), F = F statistic, *p* = *p value*, BP *p* = Breusch-Pagan *p value*, * = significance of the result, SW *p* = Shapiro-Wilk *p value*.

**Fig. 2:**
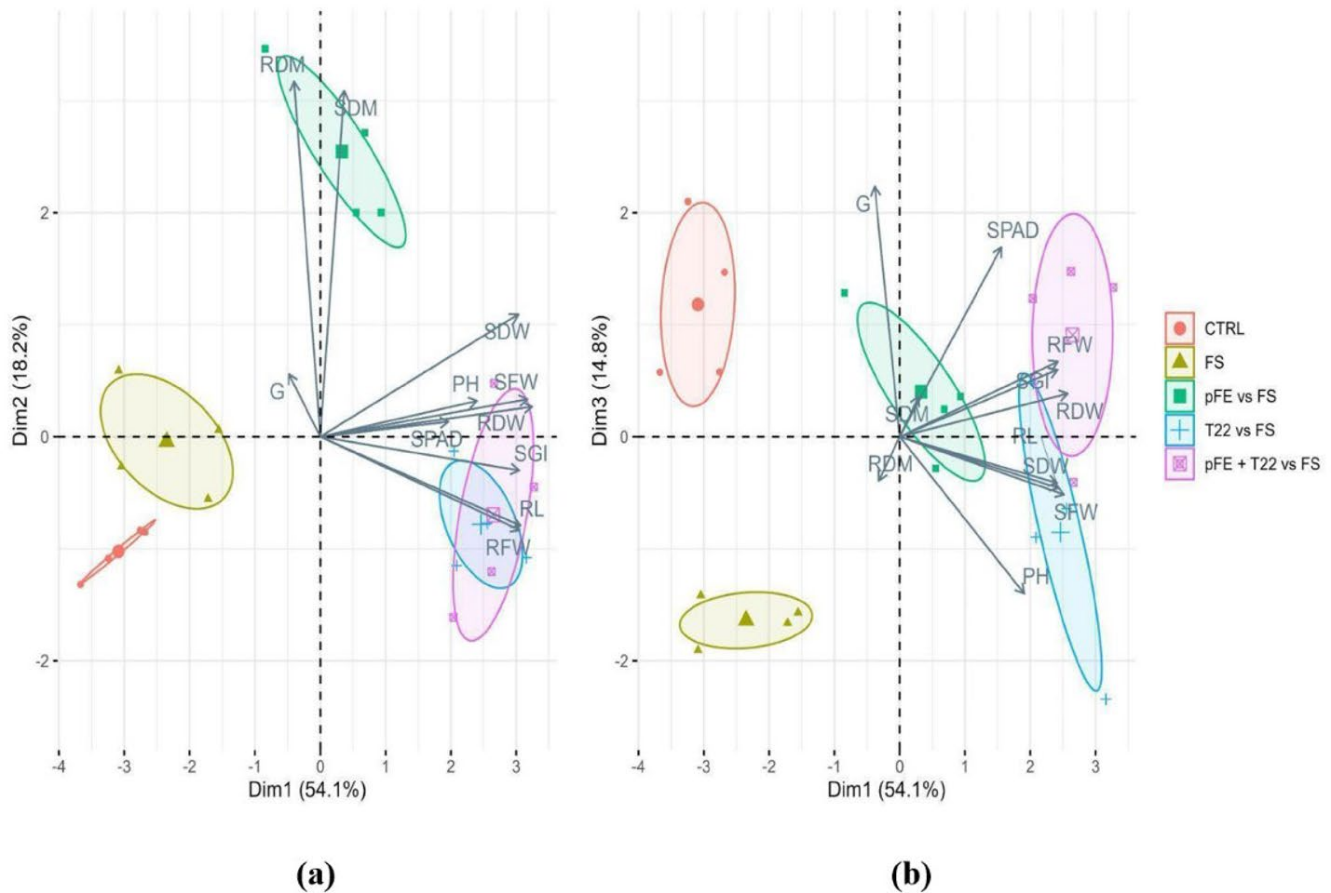
Biplots for the principal component analysis (PCA) outputs for the effect on growth parameters in durum wheat (*Triticum durum* Desf. var *Simeto*) seedlings derived from: untreated seed (CTRL) or seeds primed with frass extract (pFE), *T. afroharzianum* T22 (T22), alone or together (pFE + T22) and infected with *F. sporotrichioides* (FS). **a**: biplots of dimension 1 (Dim1) and dimension 2 (Dim2); **b**: biplots of dimension 1 (Dim1) and dimension 3 (Dim3). G = percentage of germinated seeds/total sowed seeds (%), RL = radicle length (cm), SGI = seed germination index (%), PH = plant height (cm), SPAD = index of chlorophyll content in leaf, L = leaves number, SFW = shoot fresh weight (g), RFW = root fresh weight (g), SDW = shoot dry weight (g), RDW = root dry weight (g), SDM = shoot dry matter (%), RDM = root dry matter (%)

The effect on growth parameters induced by priming with pFE, T22 and pFE + T22 on wheat seeds when these were also infected by *F. sporotrichioides* (FS) is shown in Fig. 2; Table 2. Dim1 in PCA plot accounted for 54.1% of the variance, while Dim2 for 18.2%, and Dim3 for 14.8%, so that their sum explained 87.1% of total variance. Dim1 was principally driven by RL, SGI, and fresh and dry weights of both shoot and root. Dim1 clearly separated CTRL and also FS from all the other treatments, placing them in the negative quadrant because of their significantly lower values of all these parameters, except RL (in CTRL) and RFW, than values of treatments (Fig. 2a; Table 2). In particular, the treatment with pFE (pFE vs. FS) was across the axis of Dim1 due to the negative association with high values of RL and RFW, in fact not significantly different from those of CTRL and/or FS (Fig. 2a; Table 2). Meanwhile, when used in combination with T22, the frass extract (pFE + T22 vs. FS) showed significantly higher values of RL, RFW, SDW, and RDW with respect to the CTRL and FS (Table 2), so that it was completely placed, together with the treatment T22 vs. FS, in the positive quadrant (Fig. 2a; Table 2). Noteworthy, pFE vs. FS was the only treatment fully positioned in the upper side of Dim2 (Figs. 2a). This is because of its positive association with very high values of shoot and root dry matter (Fig. 2a; Table 2), that were the driver parameters for this dimension 2. Regarding the Dim3, driven by SPAD, G and PH, with a negative relation between these two latter, a significantly higher PH value for T22 vs. FS (Table 2) compared to CTRL, while lower G values for FS and T22 vs. FS, as well as lower SPAD values for FS in contrast to CTRL and/or to all the other treatments. These values determined the position, completely or almost completely, in the negative quadrant for FS and T22 vs. FS, respectively (Fig. 2b; Table 2).

**Table 2:** Effect on growth parameters in durum wheat (*Triticum durum* desf. Var *Simeto*) seedlings derived from: untreated seed (CTRL), seeds infected with *F. sporotrichioides* (FS), and seeds infected and primed with Frass extract (pFE vs. FS) and *T. afroharzianum* T22 (T22 vs. FS), alone or together (pFE + T22 vs. FS)

Treatment	G (%)	SW *p*	RL (cm)	SW *p*	SGI (%)	SW *p*	PH (cm)	SW *p*	SPAD	SW *p*
CTRL	95.00 ± 10.00 a	0.153	12.10 ± 0.74 bc	0.564	102.30 ± 9.83 c	0.929	25.23 ± 2.22 b	0.955	37.17 ± 1.96 a	0.411
FS	75.00 ± 0.00 b	0.142	9.27 ± 1.14 c	0.949	95.38 ± 7.29 c	0.650	29.03 ± 1.40 ab	0.914	29.20 ± 2.46 b	0.354
pFE vs. FS	95.00 ± 10.00 a	0.109	12.99 ± 0.90 b	0.077	135.59 ± 10.74 b	0.101	29.28 ± 2.06 ab	0.716	38.60 ± 2.72 a	0.343
T22 vs. FS	75.00 ± 10.00 b	0.109	19.20 ± 2.88 a	0.582	155.60 ± 15.25 b	0.797	30.74 ± 2.07 a	0.381	39.00 ± 3.29 a	0.546
pFE + T22 vs. FS	95.00 ± 10.00 a	0.153	19.02 ± 1.36 a	0.859	199.87 ± 8.73 a	0.165	30.53 ± 1.41 a	0.926	40.03 ± 1.02 a	0.271
Statistics	df = 4, 15; F = 4.75; *p* = 0.01*; BP *p* = 0.468		df = 4, 15; F = 30.60; *p* < 0.01*; BP *p* = 0.691		df = 4, 15; F = 63.04; *p* < 0.01*; BP *p* = 0.668		df = 4, 15; F = 5.66; *p* = < 0.01*; BP *p* = 0.571		df = 4, 15; F = 13.10; *p* = < 0.01*; BP *p* = 0.913	

Treatment	SFW (g)	SW p	RFW (g)	SW p	SDW (g)	SW p	RDW (g)	SW p	SDM (%)	SW p	RDM (%)	SW p
CTRL	0.65 ± 0.06 c	0.785	0.11 ± 0.05 b	0.677	0.079 ± 0.007 b	0.246	0.017 ± 0.003 b	0.789	12.045 ± 0.366 b	0.326	18.00 ± 2.0 b	0.383
FS	0.48 ± 0.05 d	0.809	0.07 ± 0.03 b	0.513	0.057 ± 0.007 c	0.764	0.014 ± 0.005 b	0.578	11.601 ± 0.445 b	0.058	20.00 ± 4.0 b	0.941
pFE vs. FS	0.84 ± 0.11 b	0.837	0.14 ± 0.02 b	0.858	0.109 ± 0.013 a	0.839	0.034 ± 0.004 a	0.203	14.064 ± 1.037 a	0.808	31.00 ± 1.0 a	0.998
T22 vs. FS	1.01 ± 0.05 a	0.062	0.27 ± 0.03 a	0.515	0.112 ± 0.012 a	0.230	0.043 ± 0.004 a	0.522	12.210 ± 0.918 b	0.137	18.00 ± 2.0 b	0.862
pFE + T22 vs. FS	0.89 ± 0.05 ab	0.957	0.24 ± 0.03 a	0.689	0.118 ± 0.003 a	0.355	0.041 ± 0.007 a	0.797	11.762 ± 0.778 b	0.444	19.00 ± 2.0 b	0.765
Statistics	df = 4, 15; F = 38.22; *p* < 0.01*; BP *p* = 0.619		df = 4, 15; F = 24.10; *p* < 0.01*; BP *p* = 0.411		df = 4, 15; F = 33.63; *p* < 0.01*; BP *p* = 0.460		df = 4, 15; F = 31.37; *p* < 0.01*; BP *p* = 0.129		df = 4, 15; F = 6.93; *p* = < 0.01*; BP *p* = 0.510		df = 4, 15; F = 20.96; *p* = < 0.01*; BP *p* = 0.858	

*G * Percentage of germinated seeds/total sowed seeds, *RL*  Radicle length, *SGI *  Seed germination index, *PH* Plant height, *SPAD * Index of chlorophyll content in leaf, *SFW* Shoot fresh weight, *RFW* Root fresh weight, *SDW* Shoot dry weight, *RDW* Root dry weight, *SDM* Shoot dry matter, *RDM* Root dry matter.

Different letters indicate significant differences between values, according to one-way ANOVA followed by Tukey post-hoc test at *p* < 0.05.

Data are expressed as the mean of 4 replicates (each of 20 seeds) ± SD. df = degree of freedom (treatment, residuals), F = F statistic, *p* = *p value*, BP *p* = Breusch-Pagan *p value*, * = significance of the result, SW *p* = Shapiro-Wilk *p value*.

### Control of damping-off (DO) induced by *F. sporotrichioides* in post-emergence wheat seedlings

The effect of priming on DO disease incidence due to *F. sporotrichioides* infection is summarized in Table 3. Seeds infection with the pathogen spores caused the highest DO in the positive control (FS), with 85% of diseased seedlings. The priming treatment with pFE reduced the DO by 31%, while T22 of 44%. The combination of pFE and T22 determined a reduction of the DO of 38%, not significantly different from that of pFE and T22 used alone.

**Table 3:** Damping-off (DO) incidence caused by *F. sporotrichioides* on wheat seedlings derived from: seed primed with Frass extract (pFE) and *T. afroharzianum* T22 (T22), alone or combined (pFE + T22) and infected with *F. sporotrichioides* (FS)

Treatment	DO (%)	SW *p*
FS	85.00 ± 4.08 a	0.458
pFE vs. FS	53.75 ± 4.79 b	0.383
T22 vs. FS	41.25 ± 2.50 c	0.126
pFE + T22 vs. FS	47.50 ± 2.89 bc	0.316
Statistics	df = 3, 12, F = 165.43, *p = <* 0.01*, BP *p* = 0.716	

Data are expressed as mean (*n* = 4) ± SD.

Different letters indicate significant differences between values, according to one-way ANOVA followed by Tukey post-hoc test at *p <* 0.05; df = degree of freedom (treatment, residuals), F = F statistic, *p* = *p value*, BP *p* = Breusch-Pagan *p value*, * = significance of the result, SW *p* = Shapiro-Wilk *p value*.

### Total antioxidant activity

**Fig. 3:**
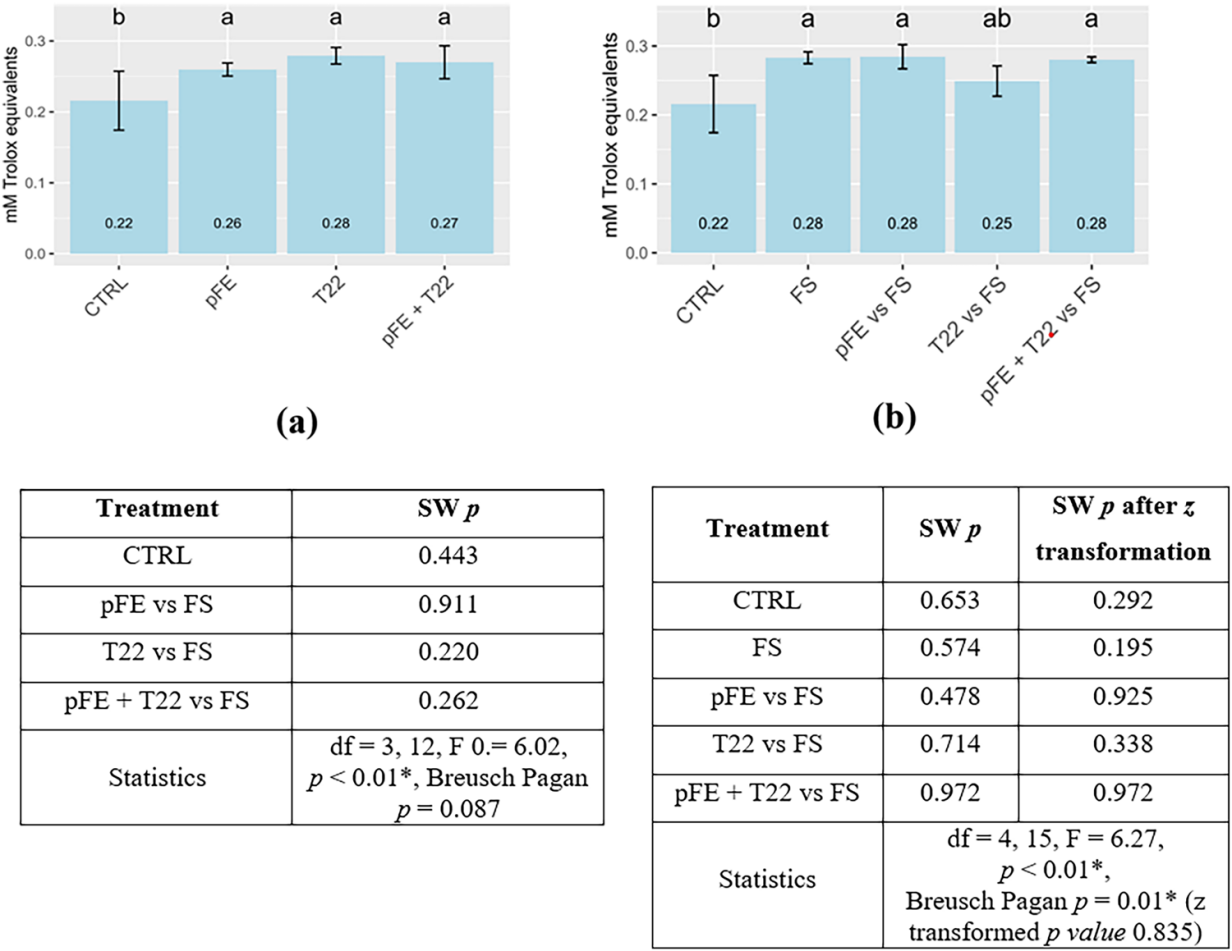
Total antioxidant activity and statistics of wheat seedlings derived from seeds primed with pFE and T22, alone or combined **(a)**; seedlings derived from seeds primed with pFE and T22, alone or combined, and also infected by FS **(b)**. Bars indicate mean values (*n* = 4) ± SD significantly different according to one-way ANOVA followed by Tukey post hoc test (*p* < 0.05). df = degree of freedom (treatment, residuals), F = F statistic, *p* = *p value*, BP *p* = Breusch-Pagan *p value*, * = significance of the result, SW *p* = Shapiro-Wilk *p value*

The antioxidant activity is reported in Fig. 3. Priming treatment caused changes in the antioxidant system of the uninfected wheat seedlings, as shown in Fig. 3a. In particular, a significant increase of the antioxidant activity was found in all plantlets derived from treated seeds compared to control (CTRL). Meanwhile, also in the presence of *F. sporotrichioides* changes in the antioxidant system were induced (Fig. 3b). Indeed, seeds infected and primed resulted in a significant increase in the antioxidant activity with respect to CTRL, except for T22 vs. FS.

### DNA extraction, PCR and sequencing

PCR reactions were successful yielding amplicons of expected sizes for each DNA barcode investigated. Nine nucleotide sequences (three/isolate and gene region) were obtained after direct sequencing. The BLASTn analysis showed a 99% similarity of the sequences obtained in this study to *Paenibacillus polymyxa* sequences already present in the NCBI database under accession numbers: ON329761.1, NR 114810.1 and NR112117.1 for the *16 S rRNA* gene; CP109848.1, CP097770.3 and CP157284.1 for the *gyrA* gene and CP133768.1 and CP139198.1 for the *rpoB* gene. All nucleotide sequences obtained in this study were deposited in the NCBI GenBank database under the following accession numbers: PV162856 and PV162858 (*16S RNA* gene); PV893198, PV893199 and PV893200 (*rpoB* gene); PV893201, PV893202 and PV893203 (*gyrA* gene).

### Phylogenetic analysis of 16 S rRNA, GyrA and RpoB genes

**Fig. 4:**
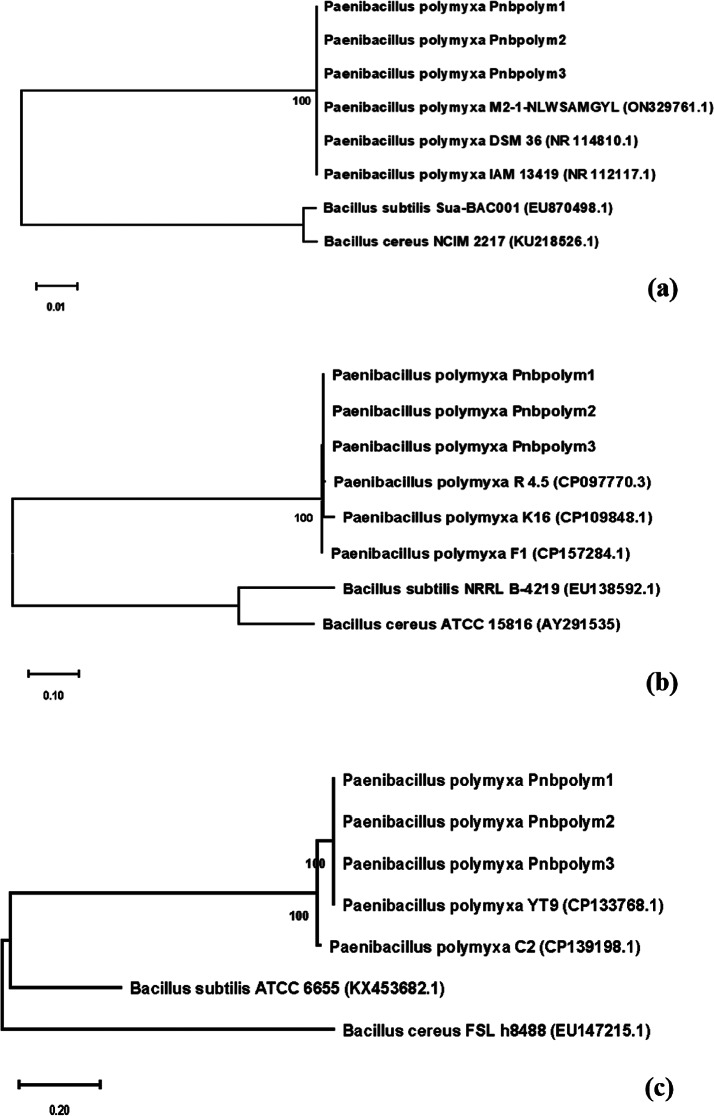
Maximum Likelihood (ML) phylogenetic trees showing the relationship of partial *16 S rRNA* (**a**), *gyrA* (**b**) and *rpoB* (**c**) barcodes of *P. polymyxa* isolates obtained in this study and other related species. Bootstrap values are indicated above the nodes. Bars 0.01, 0.10 and 0.20 represent substitutions per nucleotide site. *Bacillus subtilis* strain Sua-BAC001 (EU870498.1) and *B. cereus* strain NCIM2217 (KU218526.1) for the 16 S ribosomal RNA, *B. subtilis* strain NRRL B 4219 (EU138592.1) and *B. cereus* strain ATCC15816 (AY291535) for the *gyrA* along with *B. subtilis* strain ATCC6655 (KX453682.1) and *B. cereus* strain FLS h8488 (EU147215.1) for the *rpoB* gene were used as outgroups

Phylogenetic trees of the three barcodes investigated (*16 S rRNA*,* gyrA* and *rpoB*) showed that the bacterial isolates obtained in this study grouped together with various strains of *P. polymyxa* from different sources and origins with 100% bootstrap support while closely related *B*. *subtilis* and *B. cereus* species, taken as outgroups, clustered separately, as expected (Fig. 4: a, b and c). Furthermore, the phylogenetic data allowed to identify all bacterial isolates as *P. polymyxa* based on 100% sequence identity (for all *16 S rRNA*, *gyrA* and *rpoB* genes) with sequences of the same species already present in the GenBank. In particular, the *P. polymyxa* strains M2-1-N LWSANGYL (acc. no. ON329761.1), DSM36 (acc. no. NR114810.1) and IAM13419 (acc. no. NR112117.1) in case of the *16 S sRNA* gene; the *P. polymyxa* strains K16 (acc. no. CP109848.1), R 4.5 (acc. no. CP09770.3) and F1 (acc. no. CP157284.1) for the *gyrA* gene and the *P. polymyxa* strains YT9 (acc. no. CP133768.1) and C2 (acc. no. CP139198.1) for the *rpoB* gene found in GenBank showed identical sequences to our isolates (Fig. 4a, b and c).

### In vitro antagonistic activity induced by *P. polymyxa* isolated from frass extract against *B. cinerea*, *F. oxysporum f. sp. lycopersici* and *F. sporotrichioides*

The bacterial isolate of *P. polymyxa* (PP) found in pFE was assessed for its effects against the three phytopathogenic fungi, as summarized in Table 4. Mycelial growth was reduced by PP for all pathogenic fungi, showing the highest efficacy against BC and FOLYC (GI% of 32%) and a growth inhibition equal to 26% against FS.

**Table 4:** Percentage of mycelial growth Inhibition (GI%) of *B. cinerea* (BC), *F. oxysporum* f.sp. *Lycopersici* (FOLYC) and *F. sporotrichioides* (FS) induced by *P. polymyxa*

Fungal species	GI%	SW *p* value
BC	32.7 ± 3.5 a	0.477
FOLYC	32.4 ± 4.9 a	0.501
FS	26.4 ± 2.2 b	0.106
Statistics	df = 2, 6, F = 8.70, *p* = 0.02, Bresch-Pagan *p* value = 0.569	

Different letters indicate mean values (*n* = 3) ± SD significantly different according to one-way ANOVA followed by Tukey post-hoc test (*p* < 0.05).

## Discussion

The farming of insects generates substantial amounts of by-products, like frass, that can be valorized and advantageously used to improve crop production thanks to its richness in macro- and micro-nutrients [28, 45]. Indeed, frass can be considered as an organic fertilizer after its pasteurization by a heat treatment at 70 °C for at least 1 h, according to EC Regulation No 2021/1925 to reduce safety risk and suppress foodborne pathogens, especially *Salmonella*,* Clostridium* and Enterobacteriaceae [46]. Considering its content in microorganisms with potential plant growth-promoting activity [47] and in chitin, that could act as defense elicitor, frass can be favorably used as biofertilizer, as well as to enhance plant resilience under biotic and abiotic stress conditions, also in wheat crop [24, 48]. In particular, some Mediterranean cultivars of durum wheat released after the Green Revolution, such as Simeto, are appreciated for several important characteristics (yield potential, grain quality, tolerance to heat and drought), but they are susceptible to *Fusarium* spp [12, 13]. In our previous research [24], a first evaluation of frass extract as a possible sustainable tool for inducing biostimulant and/or antifungal activities against *Fusarium* spp. was carried out by preliminary in vitro test and in plate study on the same durum wheat cultivar used here (*Simeto*). The defined dilution of 10% pasteurized frass extract, obtained with a totally environmentally friendly method, resulted optimal for improving the growth performance and the seedling protection against *F. sporotrichioides* when used for seed priming, alone or combined with *T. afroharzianum* T22. For this reason, the same conditions were used in the current work, with the hope to gain further knowledge in the possible substitution of chemical fertilizers and/or pesticides in wheat production.

In the present study, the effect of 10% pFE, alone or combined with T22, on potted wheat seedlings derived from primed seeds, was evaluated in terms of agronomic traits, reduction of damping-off due to *F. sporotrichioides*, and activity of the pool of antioxidant enzymes involved with the aim to further unravel the possible use of insect frass and valorize the economic feasibility and circularity of the insect-rearing industry [49].

Regarding the agronomic traits, frass extract, both alone and combined with *T. afroharzianum*, also in presence of the *F. sporotrichioides* pathogen, positively influenced seedlings growth parameters. This is because all treatments determined significantly equal or higher values than untreated and not infected control (CTRL) and/or infected positive control (FS) for all considered parameters, with the exception of percentage of germinated seed (G) for pFE, pFE + T22, and T22 vs. FS, and root dry matter (RDM) for T22 (see Tables 1 and 2). This was evident from the biplots for the principal component analysis (PCA) in the biostimulation trial (see Fig. 1), where CTRL was clearly separated from all treatments and placed in the negative quadrant of dimension 1 (Dim1), that was principally driven by root length (RL), plant height (PH), shoot and root fresh and dry weights (SFW, RFW, SDW and RDW), while pFE was placed across the two quadrants because it was not positively associated only with high values of RL (see Fig. 1; Table 1). This was probably due to the property of frass from *H. illucens* to be a slow-release nitrogen fertilizer, as already described by Beesigamukama et al. 2020 [50] in a maize production. Moreover, Dim2 clearly separated both treatments with pFE (pFE and pFE + T22) from control, but also from T22, and was principally driven by G, SPAD, seed germination index (SGI) and RDM. Our results are in line with the study by Boudabbous et al. 2023 [48] where potted plants of durum wheat (variety *INRAT 100*), germinated and grown in substrate fertilizer with BSFL frass, showed height, RDW and chlorophyll indicators higher than those unfertilized. Meanwhile, the third dimension in the biplot (Dim3), principally driven by shoot dry matter (SDM) and SPAD, placed pFE completely on the negative quadrant due to the negative association with high values of both SPAD and SDM (lower values, but almost never significantly different from CTRL and/or other treatments, as shown in Table 1). This is probably because, as a slow-release nitrogen amendment, frass could promote in seedling a lower or slower accumulation of biomass in the epicotyl than hypocotyl [50]. On the other hand, SPAD and SDM resulted closely related because their relative loading vectors were almost overlapped in Dim 3 (see Fig. 2b). These parameters can be an expression of the nutritional status, being both influenced by nitrogen content in seedling [51]. Interestingly, the combination with T22 resulted in a positioning of pFE + T22 almost completely in the positive quadrant of Dim3 (see Fig. 2b), suggesting that T22 added to pFE in the priming solution could be able to improve the nitrogen use efficiency by wheat seedlings [52]. Safitri et al. (2024) [49] advised that potential solutions to improve the efficacy of frass as fertilizer are to use it combined with earthworms, or nitrification inhibitors, or mineral nitrogen fertilizers to enhance the short-term recycling of nutrients, or prevent nitrite accumulation, or to compensate eventual deficiency of readily available nitrogen by frass itself. In such a way, an improved synchronization of nitrogen release could occur thanks to a good combination between the immobilization and rapid mineralization of nitrogen, resulting in an optimal plant growth [53–57]. We can hypothesize that similar mechanisms could have been put in place when frass was used in combination with T22. *Trichoderma*-based biostimulants were already recognized as a tool to improve nitrogen use efficiency of leafy crops [52] and, therefore, T22 could be helpful for frass to improve the growth performance of seedling also in wheat.

The seed infection with *F. sporotrichioides* resulted in seedlings with values for all growth parameters equal or lower with respect to the CTRL, as expected, so that FS was placed, together with CTRL, in the negative quadrant of Dim1, driven by RL, SGI, SFW, RFW, SDW, and RDW and of Dim 3, driven by SPAD, G and PH, in PCA biplots (see Table 2; Fig. 2). Noteworthy, the presence of pFE and T22, both alone and combined, seemed to restore the growth ability in infected seedlings because pFE vs. FS, T22 vs. FS, and pFE + T22 vs. FS showed significantly higher values of these parameters with respect to FS, except for G in T22 vs. FS, PH for all thesis and RFW in pFE vs. FS (see Table 2). Effectively, this latter parameter resulted significantly equal also to CTRL, differently from what was observed during the biostimulation trial (see Table 1). This probably was due to a competition for nutrients, and following less water and nutrient uptake by pFE vs. FS, which may have established between the pathogen and the bacterium *P. polymyxa* isolated from frass extract [47]. As a consequence, pFE vs. FS was the only thesis definitely placed in the positive quadrant of Dim 2 (see Fig. 2a) because it was the only one to show the highest, and significantly different from all the other thesis, values of SDM and RDM (see Table 2). On the other hand, the presence of this beneficial bacterium in pFE could have elicited the seedling defense mechanisms based on the reinforcement of cell walls with insoluble compounds, such as synthesis of lignin, callose deposition, phenolic compounds production, in order to impede, reduce and/or prevent the penetration and spread of fungal hyphae [58, 59], as previously assessed [24]. Indeed, other than to restore the growth ability in infected seedlings, the priming with pFE ensured the significant reduction of damping-off (DO) by 31% when used alone, and almost by 40% in co-presence with T22, so strengthening the belief that some microorganisms belonging to *Bacillus* spp. and *Trichoderma* spp. have the capacity to foster plant growth and/or defense against pathogens during their life cycle, starting from seed germination [60]. Therefore, seed priming with both pFE containing *P. polymyxa* and T22 showed to be effective, at low cost and allowed the precision targeting of the pathogen *F. sporotrichioides* [61] in durum wheat *Simeto*. The reduction of DO found here coincides with that observed in the plate system from our first study [24], and it is even a little better for pFE vs. FS and pFE + T22 vs. FS (from 28% to 37% of plate system to 31% and 38% in soil, respectively). Furthermore, this DO reduction is in line with a study showing that the amendment with BSF larvae frass reduced of 30% the DO caused by *Sclerotinia minor* in garden cress [62], and of 40% the dead plants due to Fusarium wilt disease in cowpea [63].

In addition to the growth and antifungal performances, seed priming with pFE and T22, both alone and together, also led to changes in the antioxidant system. This activation of the antioxidant system in plants is responsible for triggering the synthesis of antioxidant compounds before the exposure to stress [64]. This is the reason why higher total antioxidant activity was found in seedlings derived from primed seeds even in absence of the pathogen (see Fig. 4a). The enhanced antioxidant activity allowed seedlings to face biotic stress encountered in the case of *F. sporotrichioides* infection, probably by neutralizing ROS [65]. Indeed, all seedlings derived from primed seed had enhanced total antioxidants, compared to those of CTRL, except for T22 vs. FS (see Fig. 4b). This lower antioxidant activity, similar to those of healthy seedlings, could be explained by the mycoparasitism action performed by T22 against *F. sporotrichioides*, that have determined the reduction of disease pressure, as also demonstrated by the highest DO reduction in T22 vs. FS (see Table 3) [66]. In summary, also in presence of *F. sporotrichioides*, the induction of antioxidant production due to priming with pFE and T22, both alone and combined, led to an improved tolerance by seedling against the pathogen [67]. In our previous experiment, using the same pathosystem and priming technique, we had established the defensive role of the phenolics and of superoxide dismutase (SOD), during fungal infection, as nonenzymatic and enzymatic key compounds in the possible decomposition of the fungal hyphae cell wall or in ROS detoxification [24]. In light of the interesting increased total antioxidant activity revealed here, for the first time, as seedling “alert” response thanks to seed priming with pFE, also when combined with T22, it surely will be fascinating to unravel, in future studies, the behavior of post-priming mechanisms as antifungal responses related to other antioxidant compounds counteracting ROS, such as catalase or peroxidases, in wheat under field conditions.

To better determine to which component of pFE address the antifungal activity found here, the bacterial species *P. polymyxa*, originally isolated from pFE and accurately identified based on phylogenetic data of three-locus DNA barcode, was tested for its activity against several phytopathogenic fungi. This species was able to inhibit mycelial growth of *B. cinerea* and *F. oxysporum* f. sp. *lycopersici* of 32%, while the growth reduction of *F. sporotrichioides* was about 26%. The antifungal activity of *P. polymyxa* is due to its ability to compete for nutrients, produce various antibiotics and enzymes, like fusaricidin, chitinases and glucanases, and its capacity to induce systemic resistance [68]. In agreement to our results, Zhang et al. (2021) [69] reported a similar mycelial growth inhibition of *B. cinerea*, while a slight lighter inhibition was found against *F. oxysporum*. Noteworthy, the in vitro activity of *P. polymyxa* against *F. sporotrichioides* was slightly lower to that found in vivo (DO reduced of 31%), suggesting that *P. polymyxa* was able to inhibit fungal growth similarly to the whole extract, as observed by Arabzadeh et al. (2023) [70]. The authors attributed the inhibition of fungal pathogens mycelial growth by a frass extract to the presence of microorganisms able to produce antifungal and anti-oomycetes compounds and, in particular, to the *Bacillus veleziensis* found in their extract. Further studies should be performed to verify in vivo the antifungal activity against *F. sporotrichioides* by *P. polymyxa* alone, considering that this bacterium, commonly found in soil, rhizosphere and plant tissue, is able to produce antimicrobial peptides, to solubilize insoluble phosphate, to produce indole acetic acid (IAA), as well as to degrade lignin, cellulose and hemicellulose [71, 72] efficiently used the combination of the three bacterial strains *P. polymyxa*, *Bacillus amyloliquefaciens*, and *B. subtilis* against *F. graminearum* in wheat. In agreement, the results of our study obtained from the combined use of frass, naturally containing *P. polymyxa*, and the biocontrol agent *T. afroharzianum* T22, demonstrated that this strategy can be employed for an effective control of *F. sporotrichioides* in wheat plants using the priming technique and, at the same time, was also able to promote plant growth to a greater extent.

## Conclusions

This study provided evidence on the role of frass extract as seed priming, emphasizing its capacity to positively affect agronomic traits, antioxidant and antifungal properties of wheat starting from the seed germination stage. Indeed, the interaction between pFE and the pathogen *F. sporotrichioides*, also in presence of *T. afroharzianum* strain T22, endowed wheat seedlings with an improved resilience, preparing them to effectively challenge and tolerate the biotic stress induced by the fungal pathogen. The insights gathered from this research, confirmed the possibility to use frass in priming technique, opening the door to promising solutions to harness the potential of sustainable agricultural practices and circular economy-based green technologies. Our findings support the use of natural compounds, as frass, in a cost‐efficiency contest, considering the ability of *H. illucens* larvae to feed on organic by‐products and waste. In such a way, it will be possible to reduce feed input costs and to offer a solution to managing organic waste, mitigating their impact on disposal and, at the same time, giving the wheat crop greater growth and defense performances. Although the present study positively evaluated the effectiveness of this strategy by moving from a plate system, studied during our first research, to a soil system, further studies will be necessary to confirm the performance of these seedlings in the field and during different plant phenological stages, as well as to better define molecular and biochemical mechanisms responsible of the frass extract activities. Surely, the identification of the *P. polymyxa* as a biocontrol agent further strengthens the biological relevance of the frass extract and adds value to the sustainable aspect of this strategy.

## Data Availability

The datasets used and/or analyzed during the current study are available from the corresponding author on reasonable request. The nucleotide sequences of *P. polymyxa* obtained in this study and used in the phylogenetic investigation are available from the NCBI repository under the following accession numbers: PV162856 and PV162858 (*16S RNA* gene); PV893198, PV893199 and PV893200 (*rpoB* gene); PV893201, PV893202 and PV893203 (*gyrA* gene).
